# Nonuniform Curvature and Bending Rigidity of Adsorbed Collagen Molecules in Aqueous Solution

**DOI:** 10.1021/acs.biomac.6c00845

**Published:** 2026-07-30

**Authors:** Daniela A. Barragàn Rivera, Maria P. De Santo, Elvira Brunelli, Pierluigi Bilotto, Philipp J. Thurner, Guido Raos, Bruno Zappone

**Affiliations:** † Consiglio Nazionale Delle Ricerche - Istituto di Nanotecnologia (CNR-Nanotec), via P. Bucci 33/C, Rende, Cosenza 87036, Italy; ‡ Università della Calabria, Department of Physics, via P. Bucci 31/C, Rende, Cosenza 87036, Italy; § Università della Calabria, Department of Biology, Ecology and Earth Sciences (DiBEST), via P. Bucci 4/B, Rende, Cosenza 87036, Italy; ∥ 27259 Technische Universität Wien (TU Wien) - Institute of Engineering Design and Product Development, Getreidemarkt 9, BA, Vienna 1060, Austria; ⊥ Technische Universität Wien (TU Wien) - Institute of Lightweight Design and Structural Biomechanics, Gumpendorfer Straße 7/Objekt 8, Vienna A-1060, Austria; # 18981 Politecnico di Milano, Dipartimento di Chimica, Materiali E Ingegneria Chimica “G. Natta”, via L. Mancinelli 7, Milano 20131, Italy

## Abstract

Collagen, the most abundant protein in mammals, plays a key role in tissue formation and mechanics due to its triple-helix structure. We used atomic force microscopy to study individual type-I and type-III human collagen molecules adsorbed on smooth mica surfaces from low-salt, near-neutral aqueous solutions. Statistical analysis of their two-dimensional contours revealed nonuniform curvature in both collagen types, which persisted after surface drying and molecular dehydration, owing to robust collagen–mica adsorption. In addition, the angle between tangent vectors at the ends of molecular segments followed a non-Gaussian probability distribution, indicative of nonequilibrium quenching of fluctuations upon adsorption to mica. These results suggest that collagen either possesses an intrinsic three-dimensional curvature in solution or acquires a two-dimensional curvature upon adsorption. The first scenario has implications for the self-assembly and elasticity of collagen fibrils, whereas the second has implications in biomaterial design and tissue-engineering strategies.

## Graphical abstract

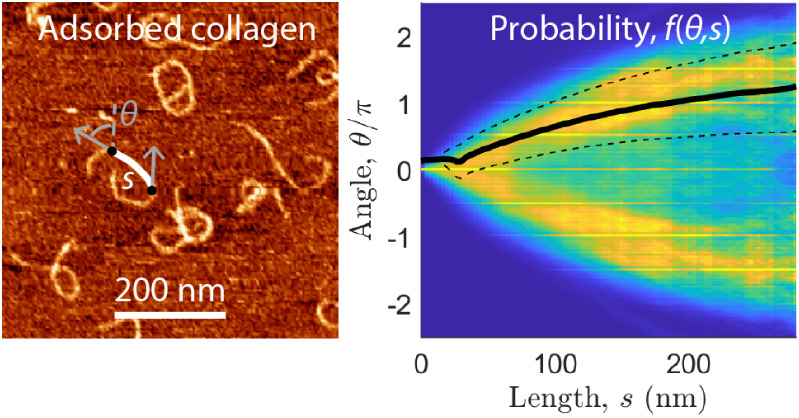

## Introduction

Collagen is the most abundant structural protein in many animals, accounting for around a third of the total protein content of the human body.^1^ It is predominantly found in connective tissues, such as bone, tendon, and skin, where it plays a fundamental role in maintaining structural integrity and mechanical resilience.^2,3^ So far, 29 types of human collagens have been identified, with fibril-forming types I and III accounting for over 90% of the collagen in the body.

The collagen molecule comprises three polypeptide chains, known as α-chains, oriented in the same direction but staggered by one residue relative to each other. The α-chains assemble into twisted triple-helix domains due to a characteristic Glycine-X-Y repeating pattern with a predominance of proline and hydroxyproline for the amino acids X and Y, respectively.^2^ In type-I and type-III collagen, the triple helix occupies most of the α-chain length, forming a stable and relatively rigid aggregate known as tropocollagen with a length of about 300 nm and a diameter of about 1.5 nm.^4^ In salt solutions at neutral pH, collagen self-assembles into highly ordered fibrils that serve as foundational components of the extracellular matrix, contributing to the load-bearing and tensile properties of connective tissues.

Due to its widespread availability as a byproduct of the meat industry, collagen is a cost-effective, renewable resource for developing biomaterials. Yet, despite the relevance to biology, medicine, and biomaterial design, the mechanical properties of collagen are not fully understood at the molecular level,^3^ particularly in comparison to other biopolymers such as DNA^5^ and filamentous actin.^6^ The case of the bending rigidity is emblematic, as values varying by more than 1 order of magnitude have been reported in the literature, depending on the collagen source, solution conditions, and experimental methods.^7^

Individual collagen molecules can be adsorbed onto smooth solid surfaces and imaged using atomic force microscopy (AFM) and electron microscopy to determine their contour length and bending rigidity from the analysis of thermal shape fluctuations.^8–13^ These studies show that adsorbed collagen exhibit an intrinsic two-dimensional (2D) curvature depending on pH and ionic strength.^7^ Collagen, however, is generally assumed to be a straight molecule based on X-ray diffraction data from collagen-mimetic protein crystals and numerical simulations.^3,4,14–17^

Permanent three-dimensional (3D) curvature and torsion of the triple-helix axis appear in collagen fibrils, where the molecules self-assemble into helical bundles around the fibril center.^18–21^ This helical coiling is currently understood as the effect of chirality on the close-packing of straight yet flexible triple-helix molecules. Crucially, the coiling of fibrils directs their self-assembly into a wavy pattern, known as crimp, which plays an essential role in the elastic response of collagen tissues.^22^ Therefore, it is imperative to clarify the origin of the intrinsic 2D curvature in adsorbed collagen molecules, as it may be related to the 3D curvature and torsion underlying the mechanical properties of fibrils and tissues. Moreover, the 2D curvature may influence the assembly and growth of collagen biomaterial at a solid interface, e.g., in tissue scaffolding and regenerative medicine.

In this article, we used AFM to measure the contour shapes of type-I and type III human collagen molecules adsorbed on mica. Since the intrinsic curvature of adsorbed molecules was observed so far only after drying the surface and dehydrating the collagen,^7^ the main goal was to assess whether curvature was caused by drying the sample. We found instead that collagen exhibits an intrinsic curvature after adsorption in aqueous solution, before drying the sample. Adsorption was rapid, irreversible, and robust. It effectively “froze” the collagen molecule in a conformation that persisted after drying and eluded simple equilibrium theories of semiflexible polymers such as the worm-like chain (WLC) model.^7^ The curvature and bending rigidity values obtained from the analysis of the molecular contour shape in aqueous solution confirmed the results obtained after drying the sample. To the best of our knowledge, this is the first AFM study of collagen molecules adsorbed and imaged in aqueous solution, advancing our understanding of collagen conformation at the single-molecule level and providing a basis for improving the design of collagen-based biomaterials.

## Materials and Methods

### Sample Preparation and AFM Imaging

Heterotrimer type-I and homotrimer type-III collagen purified from human placenta were purchased from ABCAM (Cambridge, UK, product nos . 7533 and 7535, respectively). Collagen was dispersed at a concentration of 1 mg/mL in water solutions containing 0.03 mg/mL acetic acid and 0.1 mg/mL sodium azide (for type-I) or 30 mg/mL acetic acid (for type-III). The solutions were shipped and stored at 4 °C. Less than 24 h before the experiment, the type-I collagen solution was diluted to about 1.43 × 10^–3^ mg/mL using ultrapure water (18.2 MΩ·cm resistivity, total organic carbon ≤5 ppb by Millipore-Sigma, Burlington, MA, USA). The type-III solution was diluted to a lower concentration of 1.4 × 10^–2^ mg/mL.

A round piece of muscovite mica (from JBG Metafix, Montdidier, France) with a thickness of about 0.2 mm and a diameter of 1 cm was glued onto a metal disk used as the AFM sample holder. A 35 μL droplet of collagen solution was deposited on the mica surface shortly after cleaving it with adhesive tape. After an adsorption time of 3 to 5 s, the surface was rinsed with four droplets of ultrapure water, using a Pasteur pipet to carefully remove the supernatant and avoid a direct stream of fluid at the disk center. This choice of collagen concentrations and adsorption times significantly reduced the overlap between adsorbed molecules (Figure 1).

**Figure 1:**
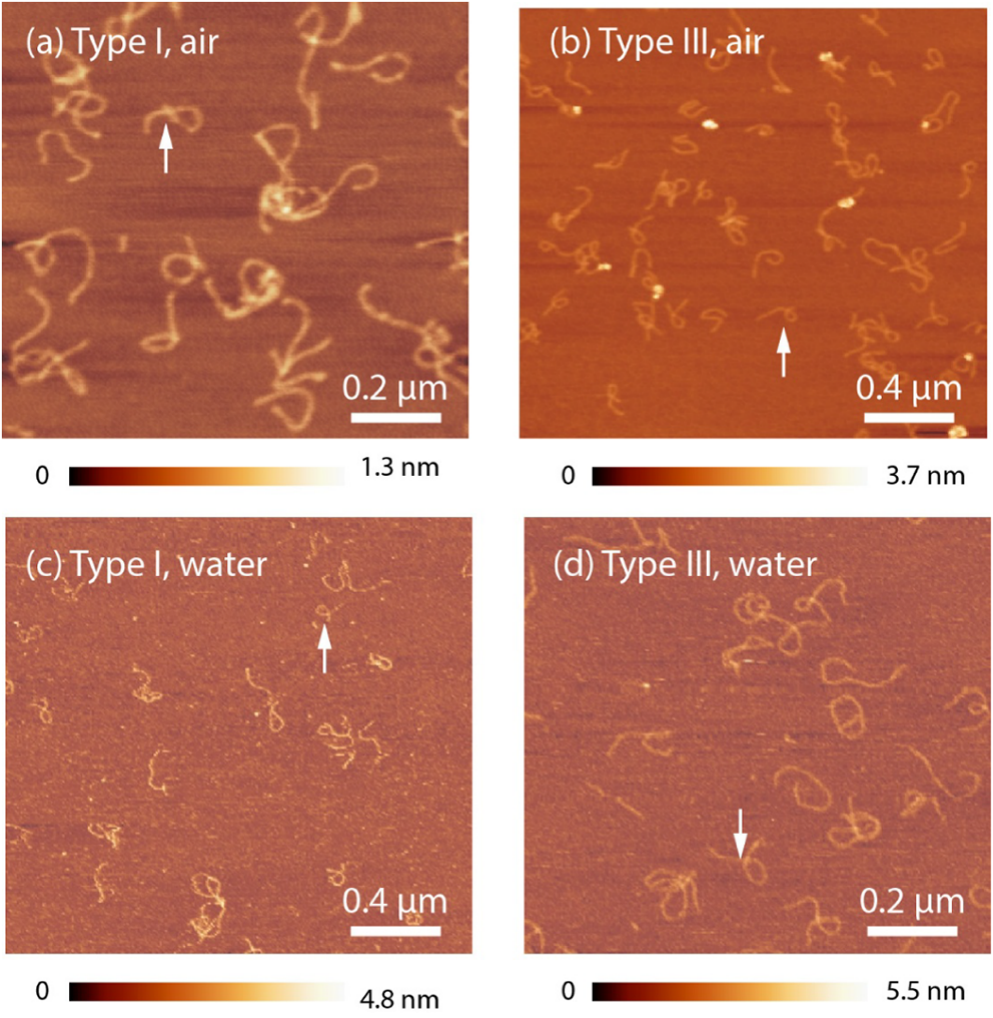
AFM images of adsorbed collagen molecules obtained in (a, b) air or (c, d) water: (a, c) type-I and (b, d) type-III collagen. The arrows show examples of self-crossing molecules.

For experiments in water, the mica surface and AFM cantilever were completely and continuously immersed in ultrapure water during the measurements. To compensate for evaporation and avoid bubble formation, water droplets were added from time to time. For experiments in air, the surface was thoroughly dried before the AFM measurements by removing water with a Pasteur pipet and drying the surface with a gentle stream of clean nitrogen (5.5 ppm purity).

We used a Multimode 8 AFM with a Nanoscope V controller (from Bruker, Billerica, MA, USA) in intermittent contact (tapping) mode. The resonance frequency and nominal spring stiffness of the cantilever probe were 150 kHz and 6 N/m, respectively for measurements in air (product no. RTESP-150 from Bruker) and 56 kHz and 0.24 N/m for measurements in water (product no. BR-DNP-S10 from Bruker). AFM images were obtained over an area of 2 μm × 2 μm following a raster scan grid with 512 × 512 points at a line rate of 1 Hz. At least three different regions of the surface were studied for each sample. For each experimental case, we repeated the measurements in four separate sessions and imaged between 90 and 120 individual collagen molecules per session.

Although collagen has an approximately circular cross-section, the apparent lateral width of a collagen molecule on the substrate was of the order of 10 nm and therefore much larger than its height *h* above the mica substrate. This artifact was due to a known shape convolution effect between the sample and the AFM tip, having a final curvature radius of the order of 10 nm.^34^

### Contour Segmentation

Adsorbed collagen molecules that did not touch or overlap other molecules on the mica surface were selected from the AFM images (Figure 1). The centerline of an adsorbed molecule was approximated as a sequence of manually selected points with in-plane coordinates *x* and *y* corresponding to pixels of the AFM image. To improve the in-plane resolution, the sequence was interpolated using a cubic spline, obtaining a smooth 2D molecular contour with subnanometer spacing between interpolation points. The height *h* of an adsorbed molecule relative to the mica substrate was determined at the in-plane coordinates of the interpolated contour using MATLAB (from MathWorks, Natick, MA, USA).

The sequence of *N* + 1 interpolated points **r**_*n*_ = (*x*_*n*_, *y*_*n*_), where *n* = 0, 1, ..., *N*, defined *N* bond vectors δ**s**_*n*_ = **r**_*n*_ – **r**_*n*–1_ with length δ*s*_*n*_ = (δ*x*_*n*_^2^ + δ*y*_*n*_^2^)^1/2^, where δ*x*_*n*_ = *x*_*n*_ – *x*_*n*–1_, δ*y*_*n*_ = *y*_*n*_ – *y*_*n*–1_, and *n* > 0. The bonds with *n* > 2 made an angle θ_*n*_ = atan­(δ*y*_*n*_/δ*x*_*n*_) with the first bond (*n* = 1), taken as the *x*-axis. The surface normal was taken as the *z-*axis, and the sign of θ_*n*_ was assigned conventionally in the right-handed *xyz* reference frame.

A pair of distinct points along the contour, i.e., with indices *p* and *q > p*, defined a curved molecular segment with contour length *s =* Σ_*k*_δ*s*_*k*_ and angle θ = θ_*q*_ – θ_*p*_ between the initial and final bond vector, where *k* = *p* + 1, ..., *q*. For each molecule, we considered all the *N*(*N* + 1)/2 possible pairs of different points *p* and *q* along the molecule, i.e., all possible molecular segments. For a molecule with total contour length *L* and an average spacing δ*s* between sampling points, there were approximately 1 + (*L* – *s*)/δ*s* segments with similar length *s* but different positions along a molecule. Since *L* ≫ δ*s* in our experiments, the segmentation produced a large number of segments per molecule, allowing a detailed study of how the angle θ depends on the segment length *s*. Specifically, we tested the hypothesis that all segments with equal length *s* were statistically equivalent regardless of whether they belonged to the same molecule (i.e., they were “cut” from different regions of the molecule) or to different molecules. In other words, we considered that the statistical distribution of the angle θ only depended on the segment length *s.*

The probability of finding an angle θ­(*s*) between the extremities of a segment with length *s* was equal to the probability of finding the angle −θ, because the orientation of the segment was arbitrary, whereas the angle changed sign when the orientation was inverted. The experimental distribution of angle, however, showed an excess of negative angles, most likely due to a psychological bias toward orienting curved objects in the clockwise direction. This bias was corrected by systematically adding the angle −θ to the statistic when the angle θ was measured for a segment length *s*.

## Results

### Adsorbed Collagen Morphology

Type-I or type-III collagen was adsorbed on freshly cleaved surfaces of muscovite mica from dilute water solutions and imaged with the AFM both in water and in air, after drying the sample (Figure 1). In all cases, most adsorbed molecules were isolated, i.e., they did not form aggregates and did not cross or overlap with other molecules.

On one hand, this observation indicates that the interaction between molecules was very weak or repulsive. On the other hand, the molecules were adsorbed flat on the substrate over their entire contour length and did not show dangling or coiled unabsorbed segments, implying a strong collagen-mica attraction. Indeed, the height *h* of a molecule relative to the mica substrate only showed small variations along the molecular contour, except at points where the molecule crossed itself (Figure 1). The average thickness *h* away from a crossing, the thickness *h*_2_ of a crossing, and the difference *h*_2_ – *h* decreased in air relative to water, showing that dehydration significantly affected the molecular structure (Table 1). The height *h* in air was smaller than 1 nm, in agreement with previous AFM measurements on mica.^9,11–13^ In water, the height was slightly larger than 1 nm and closer to the diameter of 1.5 nm obtained from collagen crystal X-ray diffraction,^4^ indicating that water molecules were embedded in the triple-helix structure. Interestingly, the height of the crossings in water was smaller than the expected value *h*_2_ = 2*h,* suggesting that the molecules flattened at the crossing point.

**Table 1:** Average Height *h* of Adsorbed Collagen Molecule Relative to the Mica Substrate Far from Crossings, and Height *h*_2_ at the Crossings^a^

	*h*	*h*_2_	*h*_2_ – *h*
**Type I, air**	0.378 ± 0.004 (9635)	0.604 ± 0.10 (39)	0.23
**Type I, water**	1.037 ± 0.011 (8452)	1.587 ± 0.26 (37)	0.55
**Type III, air**	0.518 ± 0.007 (6173)	0.961 ± 0.20 (23)	0.44
**Type III, water**	1.058 ± 0.011 (9191)	1.599 ± 0.26 (38)	0.54

a All values are given in nm with the standard error. The parentheses show the number of measurement points.

The contour length *L* of the adsorbed collagen molecules followed an approximately Gaussian distribution with average and standard deviation (255 ± 13) nm for type-I in air, (275 ± 13) nm for type-I in water, (262 ± 27) nm for type-III in air, and (280 ± 15) nm for type-III in water (Figure 2).

**Figure 2:**
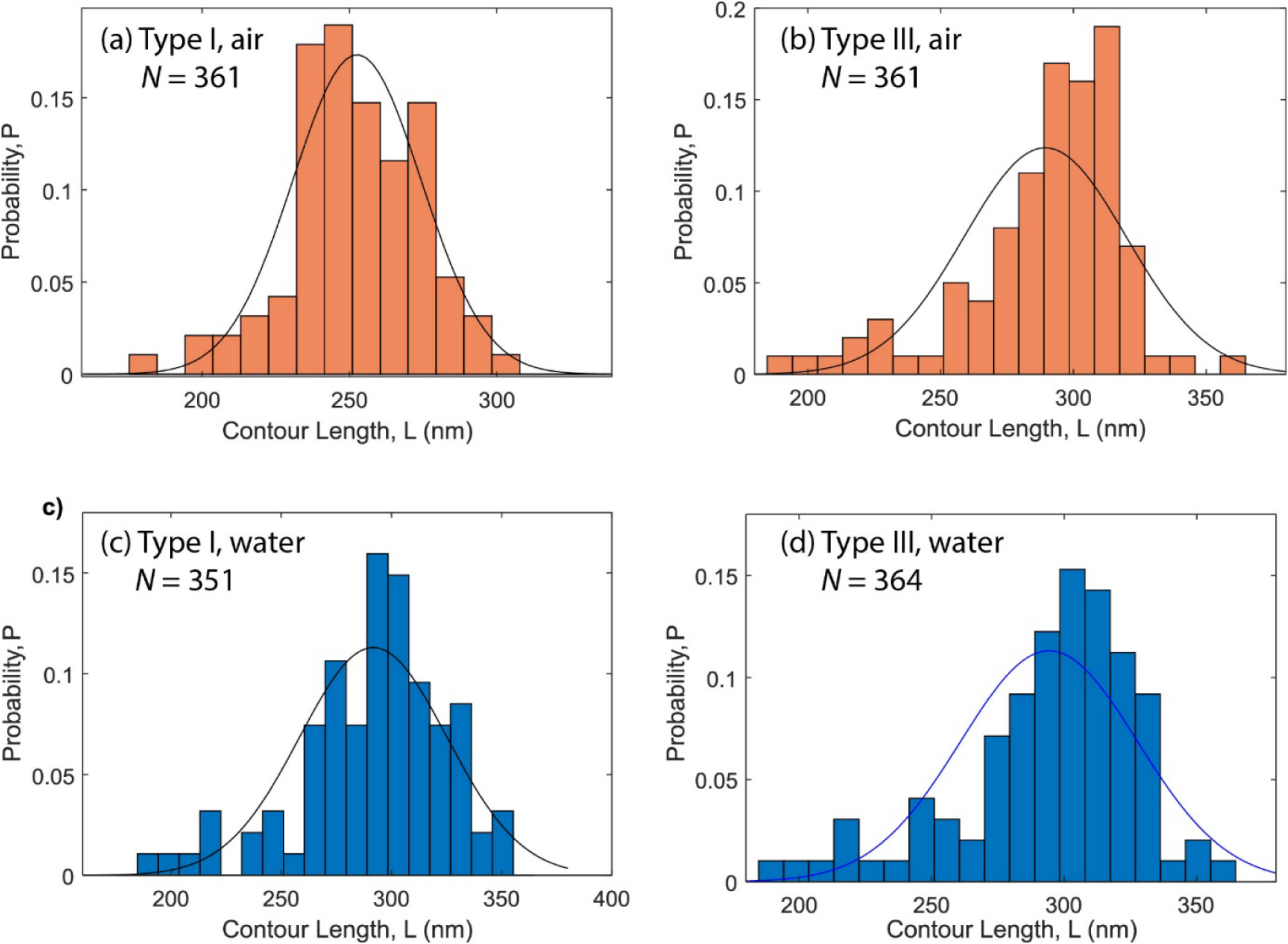
Distribution of contour lengths *L* measured for type-I and type-III collagen adsorbed on mica and imaged in (a, b) air and (c, d) in purified water. The solid lines are Gaussian fits to the experimental data. *N* is the number of molecules included in the analysis.

Analysis of variance (ANOVA) did not show any statistically significant difference among the four experimental cases. These values agree with those reported in the literature,^8,9,11–13^ and we note that a (5–10)% dispersion in contour length measurements is common for collagen as well as double-stranded DNA.^5^ The contour length analysis excluded molecules with lengths deviating by more than three times the standard error from the average. These molecules most likely were the result of collagen degradation and end-to-end association in water solution, possibly initiated at the nonhelical ends of the collagen molecule.

### Nonuniform Curvature

Adsorbed collagen molecules that did not touch or overlap other molecules on the mica surface were selected from the AFM images (Figure 1). The centerline of an adsorbed molecule was approximated as a 2D curve [*x*(*t*), *y*(*t*)] with contour length *L,* where *x* and *y* are the surface coordinates and *t* is a curvilinear coordinate with a spacing δ*s* ≈ 1 nm between neighboring points. Each curve was segmented to determine the angle θ­(*s*) between the tangent vectors at two points along the curve separated by a distance δ*s* ≤ *s* ≤ *L.* Figure 3 shows the probability of finding an angle θ for a curved segment with length *s* measured in the four experimental cases considered. Figure S1 in (Supporting Information SI) shows the complete data set based on 16 measurement sessions.

**Figure 3:**
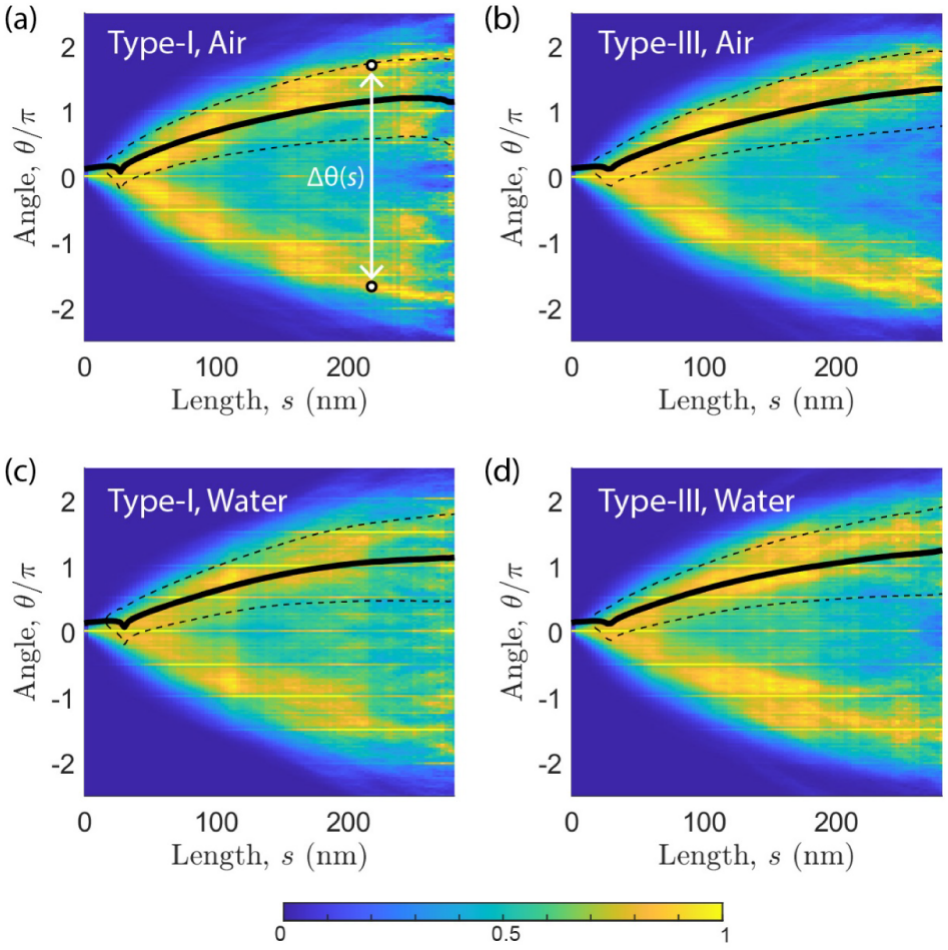
Probability of finding an angle θ between the tangent vectors at the ends of an adsorbed segment with length *s* for (a) type-I and (b) type-III collagen in air, and for (c) type-I and (d) type-III collagen in water. The color scale represents the normalized probability density *f*(*s*,θ)/*f_max_* (*s*) ≤ 1, where *f_max_* (*s*) is the maximum of *f* obtained for the length *s.* Δθ is the distance between the probability peaks obtained at a given length *s*. The thick solid and dashed black lines correspond to the angles θ_0_ and θ_0_ ± σ, respectively, of the single Gaussian calculated from the measured distribution moments <θ^2^> and <θ^4^> (eqs 2 and 3).

For lengths *s* larger than 40 nm, the probability showed two distinct peaks at angles ±θ_0_ spaced by the distance Δθ/2. The spacing Δθ between these peaks increased sublinearly as the length *s* increased, i.e., with a slope *∂*Δθ/*∂s* that decreased as the length *s* increased in all four experimental cases.

To understand this result, we approximate the adsorbed molecular contour as a sequence of short rigid bonds with length δ*s*, so that the angle between tangent vectors at the segment ends can be written as the sum of the bond angles: θ = Σ_*n*_δθ_*n*_. If the most probable angle was θ = 0, then a positive bond angle δθ_*n*_ along the segment would be equally probable as a negative bond angle. Instead, the most probable angles ±θ_0_ were different from zero, i.e., the bond angles δθ_*n*_ tended to have an equal sign along the segment. In other words, the segment tended to be curved in the *xy-*plane of the substrate. Moreover, if the most probable bond angle was constant along the segment, δθ_*n*_ = δθ, the segment would approximate an arc of a circle with an angle θ = κ*s* increasing linearly with the segment length *s*, where κ = δθ/δ*s* is the curvature. Instead, the data show that the most probable angle increases sublinearly with *s*, i.e., with a variable curvature (Figure 3).

### Non-Gaussian Probability Distribution

The worm-like chain (WLC) model is widely used to characterize thermal fluctuations and mechanical response of polymers and biomolecules with a relatively large bending stiffness, such as double-stranded DNA, actin filaments, and collagen.^7^ The molecule is treated from the thermodynamic point of view as an elastic rod that can stretch, bend, and twist around its centerline.^23^ In the absence of applied forces, thermal fluctuations that stretch and twist the molecule are usually ignored as they require a high free-energy cost. On the other hand, fluctuations can bend the molecule relative to its ground state. In the 2D version of the WLC model, the molecular shape is specified by the curvature κ­(*t*) = *d*θ/*dt* describing how the angle θ­(*t*) between the tangent vector and the *x*-axis varies along the molecule as a function of the curvilinear distance *t* from one of the molecule’s ends. If a WLC molecule is regarded as a sequence of bonds with equal length δ*s*, the probability of finding an angle δθ_*n*_ between the bonds of order *n* and *n* + 1 is given by the Gaussian distribution:

1 $$g\left(\delta \theta_{n}-\delta \theta_{n}^{0}\right)=\frac{e^{-{\left(\delta \theta_{n}-\delta \theta_{n}^{0}\right)}^{2}/2{\sigma_{n}}^{2}}}{\sqrt{2\pi }\sigma_{n}}$$

where δθ^0^_*n*_ = κ^0^_*n*_δ*s* is the average bond angle, σ_*n*_^2^ = δ*s*/*p*_*n*_ is the variance, κ^0^_*n*_ = 1/ρ^0^_*n*_ is the curvature in the ground state, ρ^0^_*n*_ is the curvature radius, and *p*_*n*_ is the persistence length, related to the bending rigidity.^7,23^ Both κ^0^_*n*_ and *p*_*n*_ generally depend on *n*, i.e., they can vary as a function of the position along the molecule.

The angle between the tangent vectors at the segment ends is given by the sum angle θ = Σ_*n*_δθ_*n*_. Because the distribution of each bond angle δθ_*n*_ is Gaussian (eq 1), the probability of the sum θ is the Gaussian function *g*[(θ – θ_0_)/σ] with average θ_0_ = δ*s*Σ_*n*_κ^0^_*n*_ and variance σ^2^ = δ*s*Σ_*n*_(1/*p*_*n*_). Therefore, WLC segments molecules with uniform curvature κ_0_ and persistence length *p* show an average angle θ_0_ = κ_0_ *s* between tangent vectors at the segment ends and variance σ^2^ = *s*/*p*, both increasing linearly with the segment length *s*. Notably, curvature only affects the average angle, whereas the persistence length only affects the variance.

In our experiments, the two ends of a collagen molecule were indistinguishable from each other, and the molecular contour could not be oriented (Figure 1). As a consequence, a segment could be sampled with equal probability in one direction or the other. The angle θ between the segment ends, however, changes sign when the sampling direction is reversed. Therefore, our measurements had equal probability of finding θ or −θ for the same segment, requiring that the distribution function *f*(θ) be a symmetric even function of θ. Namely, *f* = (1/2)­[*g*(θ) + *g*(−θ)], where *g*(θ) is the distribution that would be obtained if the segments could be oriented. In our case, therefore, the WLC model predicts a symmetric double Gaussian for the angle distribution,

$$f=\left(1/2\right)\left\{g\left[\left(\theta -\theta_{0}\right)/\sigma ]+g[\left(\theta +\theta_{0}\right)/\sigma \right]\right\}$$

where *g* is the single Gaussian distribution. The double Gaussian has an average angle <θ> = 0, variance (2nd moment) <θ^2^> = σ^2^ + θ_0_^2^, and fourth moment <θ^4^> = 3σ^4^ + 6σ^2^θ_0_^2^ + θ_0_^4^. The center θ_0_ > 0 and variance σ > 0 of the single Gaussian *g*(θ ± θ_0_) can be calculated from the second and fourth moment of the double Gaussian using simple algebra:

2 $$\theta_{0}^{2}=\sqrt{\frac{3<{\theta^{2}>}^{2}-<\theta^{4}>}{2}}$$

3 $$\sigma^{2}=<\theta^{2}>-\theta_{0}^{2}$$

Moreover, the cosine of the angle θ is given by the formula:^7^

4 $$\left\langle \cos\theta \right\rangle =\cos\left(\kappa_{0}s\right)e^{-s/2p}$$

Equation 4 shows that the average cosine is a decaying oscillating function of the length *s* when κ_0_ ≠ 0, crossing zero for the first time at *s*_0_ = π/2κ_0_ as the length *s* increases and reaching a first minimum at a length *s*_*m*_ such that tan­(κ_0_ *s*_*m*_) + 1/(2*p*κ_0_) = 0. Therefore, the curvature κ_0_ and persistence length *p* can be calculated from the lengths *s*_0_ and *s*_*m*_, namely:

5 $$\kappa_{0}=\pi /2s_{0}$$

6 $$p=-1/\left[2\kappa_{0}\tan\left(\kappa_{0}s_{m}\right)\right]$$

On simulated molecules obtained using the uniform 2D WCL with known values of κ_0_ and *p* (Figure S2a in SI), calculating these quantities from the lengths *s*_0_ and *s*_*m*_ produces an error of less than 15% (Figure S4 in SI).

Figure 3 shows that the distribution of angles θ was not the symmetric double Gaussian expected from the equilibrium 2D WLC model. Indeed, the spacing Δθ between the probability peaks was significantly larger than the distance 2θ_0_ between the centers of the hypothetical single Gaussians calculated from the distribution moments (eq 2). The sublinear increase of Δθ as a function of *s* was only qualitatively reproduced using a 2D WLC model with a nonuniform curvature κ_0_(*t*) having a maximum and nonzero average as a function of the curvilinear distance *t* (see Figure S2c in SI). The agreement was only qualitative because 2D WLC models produce a double Gaussian distribution for the angle θ (eq 1), in contrast with our findings. For instance, the angle distribution function calculated from 2D WLC models peaks at ±θ_0_ for large values of *s*, i.e., where the overlap between the Gaussians is negligible (Figure S2), and θ_0_ can be determined from the moments <θ^2^> and <θ^4^> of the calculated angle distribution (eq 2).

Other curvature models in which κ_0_ varied monotonically, had a minimum, or had zero average as a function of *t* produced a qualitatively different distribution of the angle θ (Figure S2 in SI).

In our experiments, the adsorbed collagen molecules often crossed themselves, i.e., they made short excursions from the *xy-*plane into the third (*z*) dimension (arrows in Figure 1). This observation suggests that adsorption interactions were strong enough to flatten the molecules, but did not enforce strict self-avoidance in 2D, which would eliminate self-crossing.

### Uniform WLC Approximation

The distribution of cosθ as a function of the segment length *s* showed a zero at *s*_0_ and a minimum at *s*_*m*_, similar to the 2D WLC model with uniform curvature and persistence length (eq 4). In fact, the deviations from the uniform WLC model highlighted in the distribution of θ (Figure 3) are not as evident in the distribution of cosθ and may have been overlooked in previous works.

Table 2 shows the average values and errors of the lengths *s*_0_ and *s*_m_. Although the WLC model does not accurately fit our data, particularly for small lengths *s* (Figure 4), it can be used to estimate the persistence length *p* and radius of curvature ρ_0_ = 1/κ_0_ using eqs 5 and 6. ANOVA testing did not show statistically significant differences in the values of *s*_0_ and *s*_m_ among the four cases. The average radius ρ_0_ = 53 nm and curvature κ_0_ = 0.019 nm^–1^ agree with previous AFM measurements of collagen adsorbed from purified water and imaged in air, whereas the average persistence *p* = 59 nm is significantly smaller.^7^

**Figure 4:**
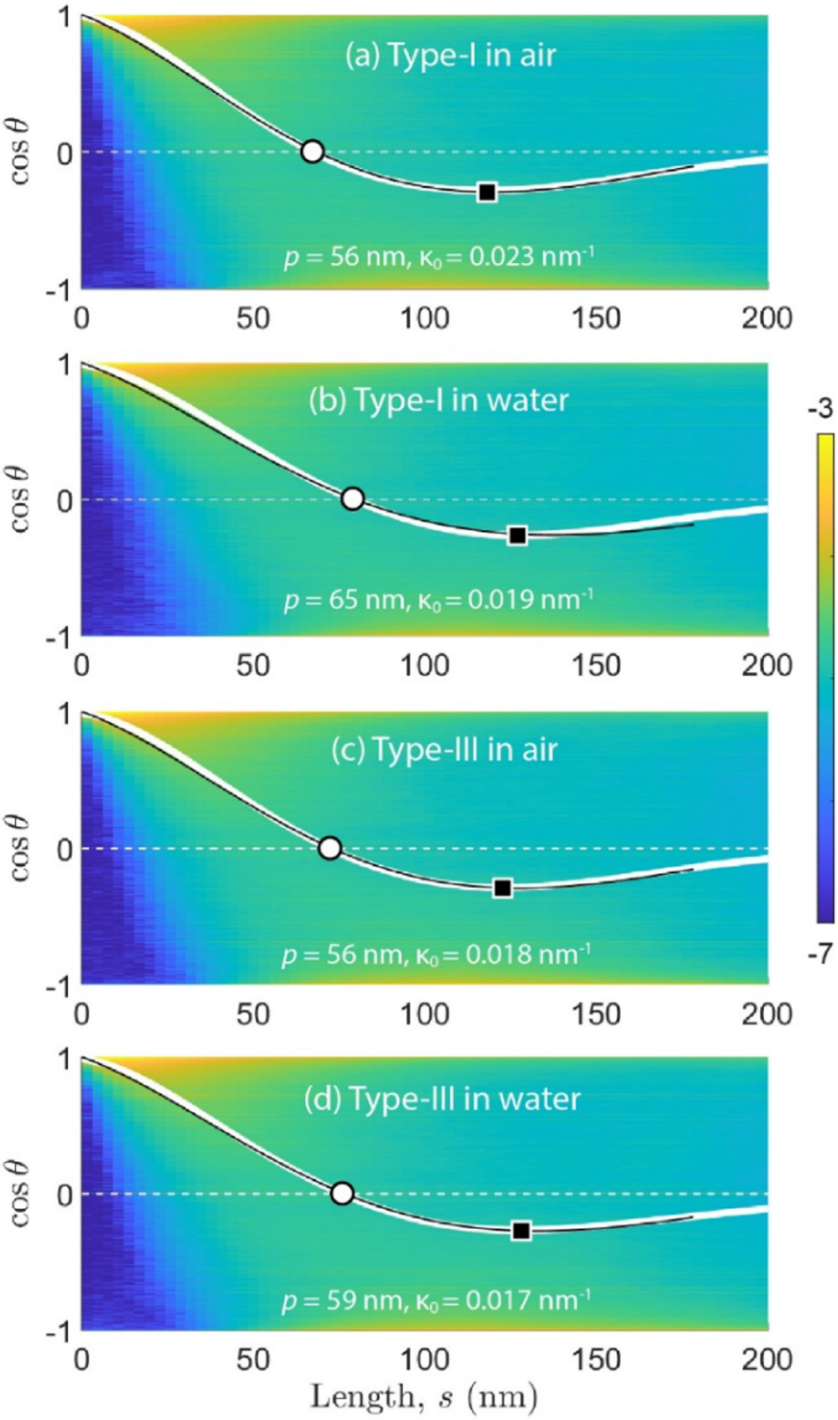
Distribution of the angle cosine, cosθ, obtained as a function of the segment length *s* for: (a, b) type I collagen and (c, d) type III collagen in air and in water. The color scale represents the logarithm of the probability density *f*(*s*, cosθ) ≤ 1. The white dot and black square show the first zero and minimum, respectively, at lengths *s*_0_ and *s*_m_. The white and black solid lines are the average <cosθ> and fit to the uniform 2D WLC model (eq 4) with values of persistence length *p* and curvature κ_0_ shown on the figures. The average values obtained are reported in Table 2.

**Table 2:** Average Values and Standard Errors of the Measured Lengths *s*_0_ and *s_m_* and Fitted Values of the Persistence Length *P* and Curvature Radius ρ_0_ = 1/κ_0_ Obtained from the Uniform WLC Model

	*s*_0_ (nm)	*s*_m_ (nm)	*p* (nm)	ρ_0_ (nm)
**Type I, air**	65 ± 6	118 ± 16	56 ±10	43 ± 6
**Type III, air**	72 ± 7	130 ± 17	56 ± 12	56 ± 4
**Type I, water**	80 ± 13	131 ± 18	65 ± 5	53 ± 9
**Type III, water**	80 ± 13	136 ± 15	59 ± 8	59 ± 8

These findings prove that surface drying and molecule dehydration did not cause curvature and, in fact, do not affect the values of κ_0_ and *p* obtained using the uniform WLC approximation.

## Discussion

Our results indicate that the adhesive interaction between collagen and mica is particularly strong in water solutions with low ionic strength and near-neutral pH, leading to the rapid, permanent, and robust immobilization of collagen on the surface. Indeed, collagen molecules were adsorbed flat on mica, and repeated AFM imaging of the same molecule in water produced the same 2D contour, showing that adsorption arrested thermal fluctuations and interactions with the AFM probe could not displace the adsorbed molecules.

Drying the sample for AFM measurements in air was expected to alter the conformation of the adsorbed molecules for various reasons. First, uneven dehydration may lead to curvature analogous to the curling of hair or plant fibers upon drying. Second, surface drying proceeds by sweeping multiple water-mica-air contact lines across the substrate, generating large capillary forces on adsorbed molecules. Third, water-soluble collagen molecules may acquire curvature by curling up around nanodroplets formed during drying. Instead, our measurements showed that the angle θ between the extremities of a segment did not significantly change as a function of the segment length *s* after drying the sample, e.g., the zeros and minimum of <cosθ> were found at the same lengths in water and in air (Table 2). Therefore, collagen adsorption on mica is particularly robust, and AFM images reflect the collagen-mica adsorption process occurring in water solution, even when AFM imaging is performed in air after drying the sample.^7^

The non-Gaussian distribution of angles θ and disagreement with the equilibrium 2D WLC model (Figure 3) also suggests that the strong and rapid adsorption effectively quenches or “freezes” collagen on mica, creating a nonequilibrium contour distribution. This effect was observed in AFM studies on double-stranded DNA, to which the 2D WLC model only applies as long as the molecules are allowed to freely fluctuate and thermally equilibrate in 2D before being immobilized on the surface.^5^ On the other hand, strong and rapid adsorption tends to create a projection of the equilibrium 3D molecular contour on the surface and creates another Gaussian contour distribution.^5^ In our experiments, therefore, the adsorption had an intermediate strength that did not produce either of the Gaussian distributions. The likely cause of the strong interaction between collagen and mica in water is electrostatic attraction, as recently suggested by direct force measurements on adsorbed collagen molecules.^24^ Indeed, mica is negatively charged,^25^ whereas collagen has a positive charge in neutral solution, due to its high isoelectric point, pI > 8.^26,27^ This charge, however, is spread throughout the collagen length with a linear charge density of about +0.17 *e*/nm (prediction based on the amino acid sequence, without taking into account post-translational modifications),^28^ in contrast to highly charged polyelectrolytes like DNA with charge density exceeding 1 *e*/nm. Additionally, collagen has heterogeneous electrostatic domains in the triple helix.^29^ Therefore, localized electrostatic attractions may work in conjunction with other surface interactions such as hydrogen bonding.

The origin of the observed 2D curvature, however, remains unclear. On one hand, the shape of adsorbed molecules may reflect an intrinsic 3D curvature of the collagen molecule, which has been recently suggested in a study on collagen-mimetic proteins in solution.^30^ On the other hand, the 2D curvature may be caused by the adsorption of triple-helix molecules that are straight yet flexible and chiral. Indeed, adsorption-induced 2D curvature has been observed for amyloid fibrils adsorbed on mica,^31,32^ sharing with collagen a twisted multistrand molecular structure. Upon adsorption, the fibrils are forced to untwist by strong surface interactions. As evidenced by helical double-stranded DNA in solution,^33^ twist and bend are inherently coupled in a WLC molecule, allowing for the exchange of energy costly twist deformations with relatively soft bending deformations. Independent of its origin, it has already been shown that the 2D curvature decreases with pH and salinity of the collagen solution,^7^ indicating that electrostatic interactions play an important role in determining the structural and mechanical properties of collagen molecules and/or their surface adsorption.

## Conclusions

In conclusion, this work provides strong experimental evidence of nonuniform intrinsic 2D curvature for collagen molecules of type-I and type-III adsorbed on a solid surface in aqueous solution. This curvature could be related to the 3D curvature of collagen molecules in self-assembled collagen fibrils, which is an essential structural feature underlying the elastic response of collagen tissue. If collagen is a straight yet flexible molecule, 2D and 3D curvature appear during surface adsorption and close-packing into fibrils, respectively, as a consequence of the triple-helix chirality. This effect could then be leveraged in the study and fabrication of collagen interfaces, particularly in medicine and biomaterial engineering, where collagen is often found in contact with artificial nonorganic materials (e.g., tissue scaffolds, implants). On the other hand, the 2D curvature of adsorbed molecules may reflect an intrinsic curvature of the collagen molecule, with profound implications in collagen fibrillogenesis. Further experiments and validation with molecular dynamics simulations are necessary to clarify this point, e.g., collagen could be adsorbed on a substrate other than mica, with different surface chemistry and electrical charge, to vary the adsorption strength and assess whether the 2D curvature persists, varies, or disappears.

## Supplementary Material



## Notes

A visual summary of the full experimental data set organized by measurement sessions; computer simulations of the angle distribution as a function of the segment length for WLC models with nonuniform curvature and uniform persistence length; and accuracy analysis for persistence length and curvature measurements based on the uniform WLC model (PDF)
