# Supplementary material for: Nonuniform Curvature and Bending Rigidity of Adsorbed Collagen Molecules in Aqueous Solution
Source: Biomacromolecules. 2026 Jul 30;27(8):5538–45. doi: 10.1021/acs.biomac.6c00845 (PMC13463538; doi:10.1021/acs.biomac.6c00845)
Supplement: Supplementary file 1 [file bm6c00845_si_001.pdf]

Supporting Information for

Non-uniform curvature and bending rigidity of adsorbed collagen molecules in aqueous solution

Daniela A. Barragàn Rivera,[1,2] Maria P. De Santo,[1,2] Elvira Brunelli,[3] Pierluigi Bilotto,[4] Philipp Thurner,[5] Guido Raos[6] and Bruno Zappone[1]

[1] Consiglio Nazionale delle Ricerche - Istituto di Nanotecnologia (CNR-Nanotec), Via P. Bucci 33/C, 87036 Rende (CS), Italy

[2] Università della Calabria, Department of Physics, Via P. Bucci 31/C, 87036 Rende (CS), Italy

[3] Università della Calabria, Department of Biology, Ecology and Earth Sciences (DiBEST), Via P. Bucci 4/B, 87036 Rende (CS), Italy

[4] Technische Universität Wien (TU Wien) - Institute of Engineering Design and Product Development, Getreidemarkt 9, BA, 1060 Vienna, Austria

[5] Technische Universität Wien (TU Wien) - Institute of Lightweight Design and Structural Biomechanics, Gumpendorfer Straße 7 / Objekt 8, A-1060 Vienna, Austria

[6] Politecnico di Milano, Dipartimento di Chimica, Materiali e Ingegneria Chimica “G. Natta”, via L. Mancinelli 7, 20131 Milano, Italy

Supplementary information Text

S1. Extended data set

Figure S1 summarizes all the results obtained for type-I and type-III collagen molecules adsorbed on mica from water solution and imaged with the AFM in air or in water. For each experimental condition (*i.e.*, type-I in air, type-I in water, type-III in air, and type-III in water), we conducted four separate sessions. We considered at least 90 AFM images of individual collagen molecules for each session, obtained from different mica surfaces.

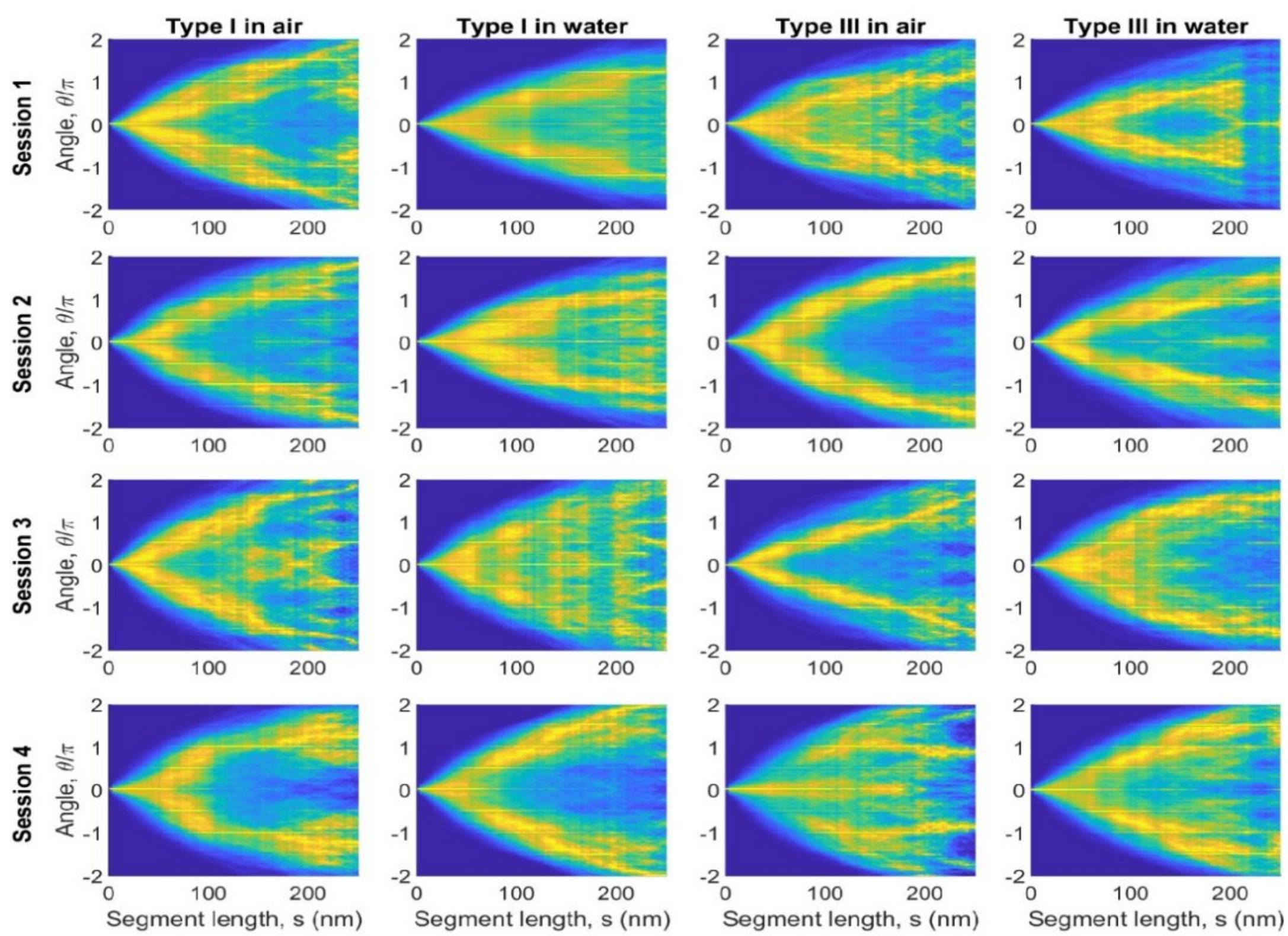

Figure S1. Probability of finding a misalignment angle θ between the ends of an adsorbed collagen segment with length s. The figure summarizes all experimental results obtained by absorbing type-I or type-III collagen on mica from water solution and imaging by AFM in water or in air.

S2. WLC model with non-uniform curvature

We used the curved 2d WLC model to simulate collagen molecules adsorbed flat on the xy-plane of the mica surface.[1] A molecule was approximated as a sequence of 280 short, straight, and rigid bonds with length $\delta s = 1$ nm and total contour length $L = 280$ nm. The first bond made a random angle with the x-axis, whereas the bond angle $\delta\theta_n$ between the bonds of order n and $n + 1$ was drawn at random from the Gaussian distribution predicted by the WLC model (Eq. 1) with prescribed persistence length p_n and curvature κ_n. Note that both these parameters could vary along the WLC molecule as a function of the bond order n. We used MATLAB to simulate 90 WLCs and segmented each molecule to obtain the distribution of the end misalignment angle θ as a function of the segment length s.

Figure S2 shows the results obtained for WLC molecules with non-uniform intrinsic curvature κ_0. We considered various models with a mean value of the curvature $\kappa_a = \int \kappa_0 ds_0 = 0.022$ nm^{-1}, corresponding to a curvature radius of $\rho_a = 1/\kappa_a = 45$ nm, and variation $\Delta\kappa = \kappa_a$. The model with minimum curvature at the center of the molecule (Fig. S2c) most closely reproduced the experimental data (Fig. 3).

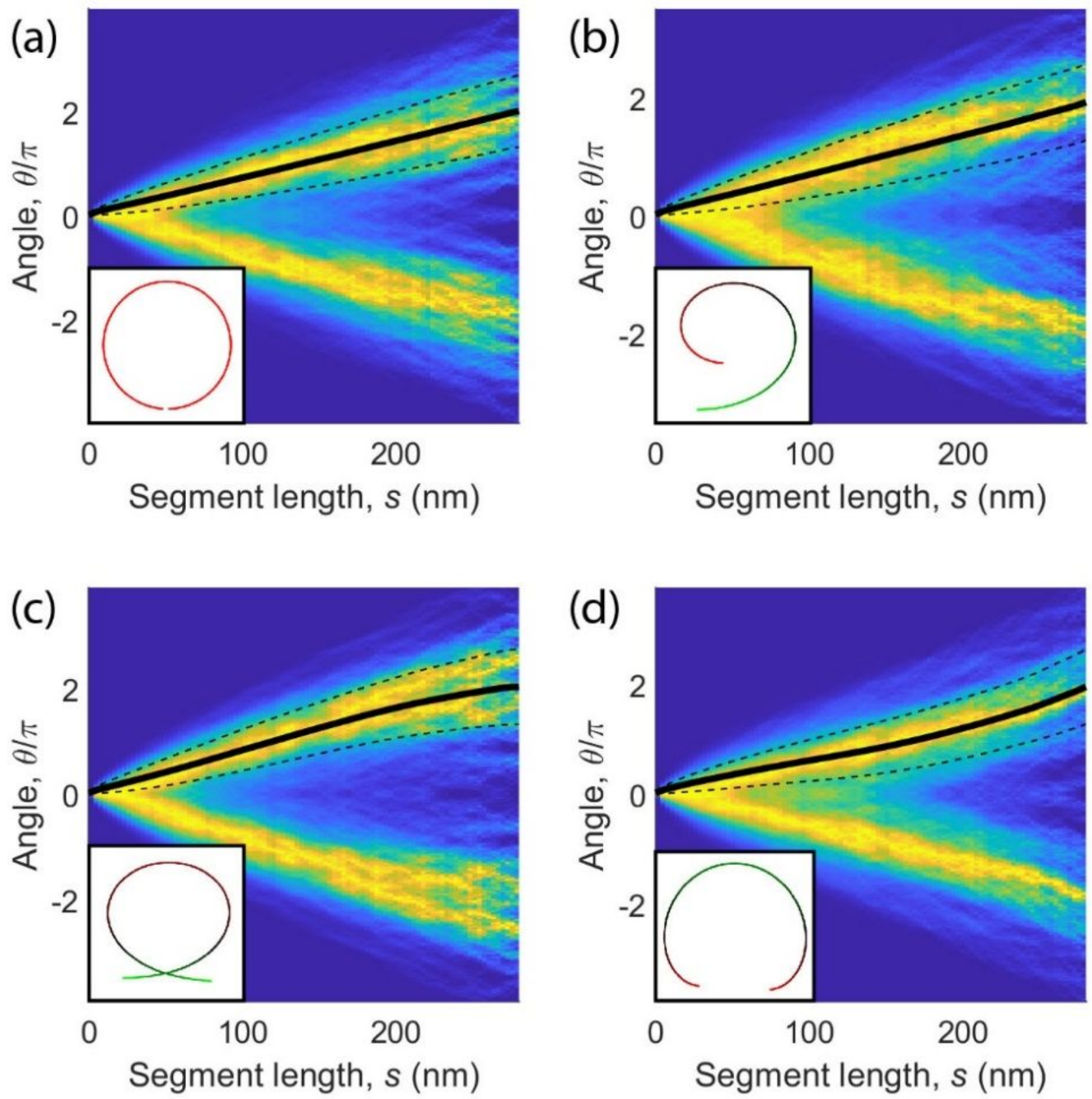

Figure S2. Misalignment angle θ between the ends of a segment with length s for simulated WLC molecules with a contour length L = 280 nm, persistence length p = 65 nm, and non-uniform intrinsic curvature $\kappa_0 > 0$ with fixed mean $\kappa_m = 0.022$ nm^{-1} (mean curved radius ρ_m = 45 nm) and total variation $\Delta\kappa = \kappa_m$. The ground-state shape of the molecule is shown in the insets, where black, green, and red correspond to curvature $\kappa_0 \approx \kappa_m$, $\kappa_0 < \kappa_m$, and $\kappa_0 > \kappa_m$, respectively. The line color is scaled as $\kappa_0/\max(|\kappa_0|)$. (a) Uniform curvature. (b) Linearly varying curvature. (c) Curvature maximum at the molecule's center. (d) Curvature minimum at the molecule's center. In each figure, the distribution was obtained from the segmentation of 90 simulated WLC molecules. The thick black line is the average angle θ_0 of the single Gaussian distribution, whereas the thin dashed

lines represent $\theta_0 \pm \sigma$, where σ is the standard deviation of the single Gaussian distribution (see Eqs 2 and 3).

The simulations in Fig. S3 were obtained by setting the mean curvature to zero, $\kappa_a = 0$, while keeping the same non-zero variation $\Delta\kappa = (1/45)\ \mathrm{nm}^{-1}$ as in Fig. S2. The zero-curvature in Fig. S3 did not reproduce the main experimental finding that there are two peaks spaced by $\Delta\theta$ in the probability of finding a misalignment angle θ (Fig. 3).

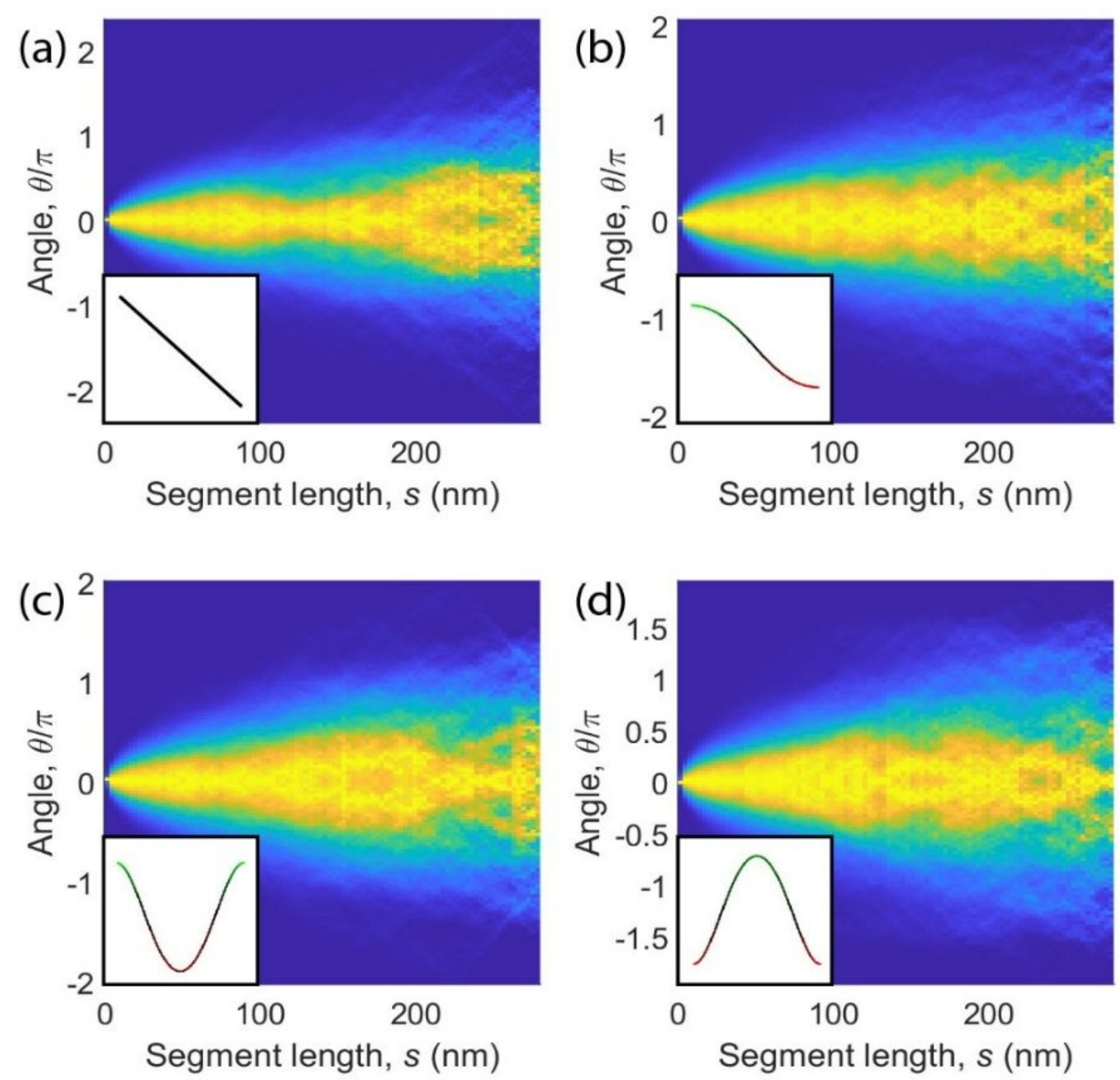

Figure S3. Variation θ of the tangent angle as a function of the segment length s for WLC molecules with a contour length L = 280 nm, persistence length p = 65 nm, and non-uniform intrinsic curvature κ_0 with zero mean κ_m and total variation $\Delta\kappa = 0.022\ \mathrm{nm}^{-1}$. The ground-state

shape of the molecule is shown in the insets, where black, green, and red indicate curvature $\kappa_0 \approx 0$, $\kappa_0 < 0$, and $\kappa_0 > 0$, respectively, and the colorbar is scaled as $\kappa_0/\max(|\kappa_0|)$. (a) Uniform curvature. (b) Linearly varying curvature. (c) Positive curvature maximum of curvature at the molecule's center. (d) Negative curvature minimum at the molecule's center. The distribution was obtained from the segmentation of 90 WLC molecules.

S3. Validation of the persistence length and curvature measurement

To determine the effect of our limited sampling of the statistical distribution of bond angles, we simulated the collagen molecules using the uniform 2d WLC model with non-zero curvature (Fig. S2a).[23] For a given persistence length p and curvature radius ρ_0, we generated 90 molecular contours and performed the same analysis as in the AFM experiments. Namely, we segmented the contours to analyse the distribution of the misalignment angle θ as a function of the segment length s (Fig. 3 and 4), and repeated the simulation in four different sessions (Fig. S1). From the distribution of the cosine of the misalignment angle, $\cos\theta(s)$, we determined the positions s_0 and s_m of the first zero and maximum, respectively, and calculated p and ρ_0 as explained in the main article (Eqs 5 and 6) with an error of less than 15% (Fig. S4).

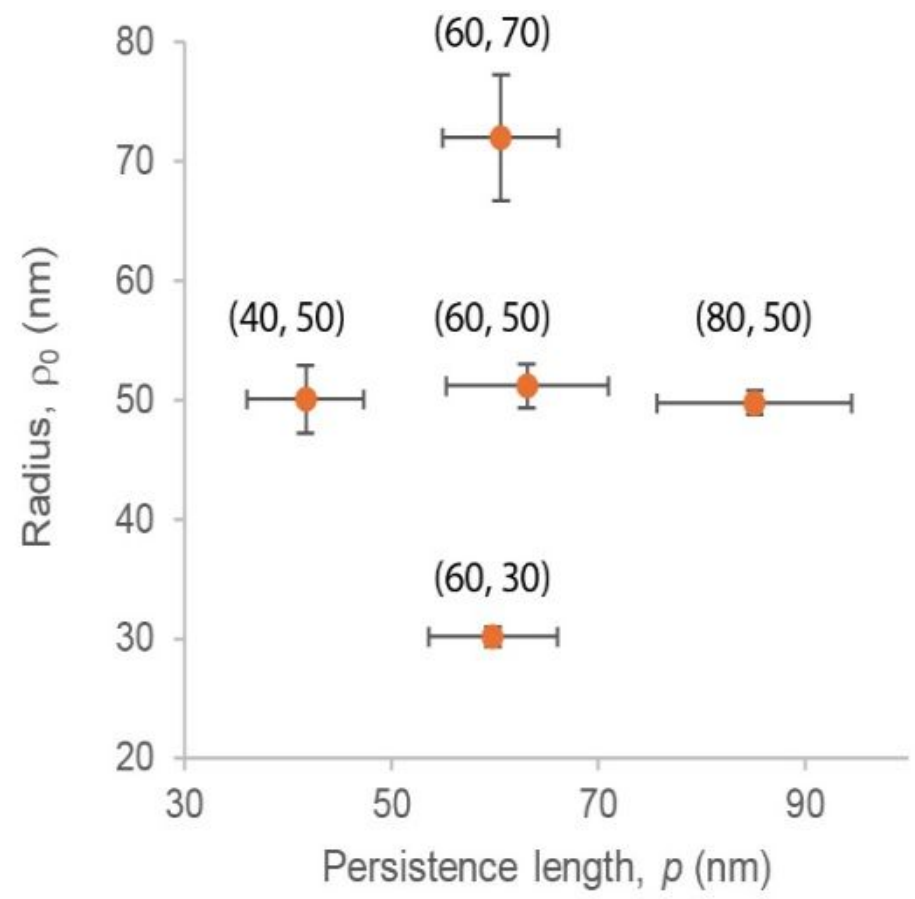

Figure S4. Persistence length p and curvature radius ρ_0 obtained from the statistical analysis of 90 contour lengths simulated using the uniform 2d WLC model. Each dot represents the average of four different simulation sessions using the same prescribed values of (p, ρ_0), indicated in parentheses above each dot. The error bars represent the dispersion ranges of measured values, from minimum to maximum.

REFERENCES

1. Rezaei, N., Lyons, A. & Forde, N. R. Environmentally controlled curvature of single collagen proteins. *Biophysical Journal* **115**, 1457–1469 (2018).
